# Efficacy and Safety of Intra-Articular Cross-Linked Sodium Hyaluronate for the Treatment of Knee Osteoarthritis: A Prospective, Active-Controlled, Randomized, Parallel-Group, Double-Blind, Multicenter Study

**DOI:** 10.3390/jcm12082982

**Published:** 2023-04-19

**Authors:** Tomasz Blicharski, Piotr Łukasik, Rafal Plebanski, Zbigniew Żęgota, Marek Szuścik, Erik Moster, Karel Pavelka, Seonhui Jeon, So La Park

**Affiliations:** 1 Clinic of Rehabilitation and Orthopedics, Medical University of Lublin, 20-090 Lublin, Poland; 2 Trauma and Orthopedic Ward, NZOZ Medi-Spatz, 44-100 Gliwice, Poland; lukasikp@poczta.onet.pl; 3 Clinic of Healthy Bone, 90-552 Lodz, Poland; osteoporoza.leczenie@wp.pl; 4 Specjalistyczny Osrodek Leczniczo Badawczy (Specialist Treatment and Research Center), 14-100 Ostroda, Poland; zzegota@tlen.pl; 5 Orthopedic Department, Rydygier’s Hospital, 31-826 Krakow, Poland; szuscik.m@gmail.com; 6 Rheumatic Center of Dr. Mostera, 61500 Brno, Czech Republic; emoster@revmacentrum.cz; 7 Institute of Rheumatology, 12850 Prague, Czech Republic; pavelka@revma.cz; 8 Life Sciences, LG Chem, Ltd., Seoul 07336, Republic of Korea; seonhuijeon@lgchem.com (S.J.); pphhoo@lgchem.com (S.L.P.)

**Keywords:** viscosupplementation, intra-articular hyaluronic acid, knee, therapeutics

## Abstract

The safety and efficacy of Hyruan ONE (test product), an intra-articular cross-linked sodium hyaluronate injection, to treat mild-to-moderate knee osteoarthritis was compared with that of Durolane (comparator) in a prospective, active-controlled, parallel-group, double-blind (masked-observed), multicenter non-inferiority study. European patients (*n* = 284) were randomized 1:1 (test product:comparator) and received one injection of cross-linked hyaluronic acid (60 mg/3 mL). In total, 280 patients completed the study. The primary endpoint of mean change in Western Ontario and McMaster University (WOMAC)–Likert Pain sub-scores from baseline at week 13 revealed changes of −5.59 and −5.54 for the test and comparator groups, respectively, demonstrating non-inferiority of the test product (difference, −0.05 [95% confidence interval, −0.838 to 0.729]). Secondary endpoint results, which included changes in WOMAC–Likert Pain sub-score from baseline to 26 weeks post-injection and changes in WOMAC–Likert Total score and Physical Function and Stiffness sub-scores, changes in patients’ and investigators’ global assessments, use of rescue medication, and responder rates at 13 and 26 weeks post-injection were similar between the groups. Incidence of adverse events was also similar. In both groups, most treatment-emergent adverse events were mild/moderate. Hyruan ONE was non-inferior to the comparator at 13 weeks post-injection in European patients with mild-to-moderate knee osteoarthritis.

## 1. Introduction

Osteoarthritis (OA) is challenging to treat. Though there are many treatment modalities for this disease, these are mostly symptomatic therapy options [1,2]. In cases of low-grade OA, pain management and lifestyle changes are often used, including patient education and awareness, physical therapy, and rehabilitation aids. In more severe cases, pharmacological interventions such as analgesic therapy (opioids if necessary), non-steroidal anti-inflammatory drugs (NSAIDs), steroidal anti-inflammatory drugs, symptomatic slow-acting drugs, and preparations for topical administration can be used. Depending on severity, surgical intervention with joint replacement may be required [3].

An alternative treatment option for OA is the injection of hyaluronic acid (HA) into affected joints to restore the viscoelasticity of the joint via supplementation of the synovial fluid [4,5,6]. HA is a ubiquitous glycosaminoglycan present in human synovial fluid, synovial membrane, and cartilage [7], and there is abundant evidence indicating that HA helps patients with OA [4,8]. Hyaluronate is an important component of synovial fluid, and injection of HA could potentially protect the articular cartilage and soft-tissue surfaces from trauma during joint function [9]. Thus, when HA is injected into OA-affected joints to improve the viscoelasticity of the synovial fluid, it consequently restores the lubricating and shock-absorbing properties in the joint. This process, termed exogenous viscosupplementation, can also stimulate an increase in endogenous HA levels [10,11]. Furthermore, the injection of intra-articular HA can promote proteoglycan and glycosaminoglycan synthesis within the cartilage (including endogenous HA). According to Altman et al., this decreases cartilage catabolic activities, which in turn reduces chondrocyte apoptosis and increases chondrocyte proliferation, suppressing proinflammatory mediators and inhibiting the action of pain mediators [11].

Despite a considerable amount of literature on viscosupplementation, current guidelines neither advocate for nor oppose HA injection, despite it representing a viable treatment option if other, more conservative options fail [12]. Some guidelines, such as those from the American Academy of Orthopedic Surgeons (AAOS), currently do not recommend the use of HA; however, this is suggested to be in part due to a lack of generalized results [12]. Recent evidence shows that HA may be beneficial in some patient subsets with OA [4,13]. Indeed, a randomized, multicenter, double-blind, placebo-controlled trial demonstrated significant improvements across multiple outcomes in patients with knee OA following a single injection of the HA Synvisc One compared with placebo [14].

Two recent systematic reviews showed that viscosupplementation was as effective as some NSAIDs, with more prolonged effects in some instances [4,13]. Intra-articular HA administration appears to be of benefit when other non-operative options are ineffective [13,15,16]. Conflicting evidence in the literature may be explained by the type of HA used, as only high molecular weight (HMW) HA has been shown to have a greater effect than non-selective NSAIDs and selective cyclooxygenase 2 inhibitors when used to treat knee OA [17]. Furthermore, despite providing sustained relief from knee OA symptoms [18], HMW HA for OA of the knee has only a moderate strength recommendation in the AAOS guidelines [19].

The cross-linking of linear HA increases its stability and viscosity, leading to delayed drug degradation in the body and extending the durability of a single injection [10,19,20,21]. Multiple injections are required when HMW HA that has not been cross-linked is used, and as such, most of the intra-articular HA preparations in use today are based on a multiple-injection dosing regimen [7]. These HA preparations also differ in their product characteristics, e.g., posology, volume injected, HA origin, concentration, molecular weight, and structure, including the degree of HA cross-linking. However, there is no consensus on the significance of these differences [7].

Hyruan ONE (LG Chem, Ltd., Seoul, South Korea) is a recently developed HMW, cross-linked, non-animal HA that has been shown to be effective and safe in South Korean patients with symptomatic knee OA [22,23,24]. Durolane (Q-Med AB, Uppsala, Sweden) is a similar cross-linked sodium hyaluronate injection therapy. Both are 1,4-butanediol diglycidyl ether cross-linked HAs, although the test product in this study, Hyruan ONE, has a viscoelasticity that is closer to the synovial fluid of healthy individuals. Durolane has been shown to provide sustained improvement in measures of pain, analgesic use (i.e., reduction in analgesic use), and physical function in patients with mild-to-moderate knee OA and is demonstrated to be non-inferior to Synvisc One [25]. Given that Durolane is a mainstay of treatment for OA in Europe, it was necessary to conduct a study to establish the non-inferiority of Hyruan ONE to Durolane to support its approval in Europe. Thus, the present study aimed to compare single injections of Hyruan ONE and Durolane in European patients to collect data from a European population.

## 2. Materials and Methods

### 2.1. Patients

Patients aged ≥ 40 years and diagnosed with mild-to-moderate OA based on radiographs (Kellgren–Lawrence scale Grade II–III) taken within 12 months of commencement of the study were included. The OA diagnosis had to fulfill the American College of Rheumatology criteria [26]. Patients could have OA in both knees, but the target knee for treatment was based on meeting the inclusion criteria of pain according to a Western Ontario and McMaster Universities (WOMAC)–Likert Pain sub-score of 7–17 at the screening and baseline visits and a Pain sub-score of 2–3 while walking on a flat surface. Patients also had to be able to provide consent and comply with all study requirements.

Patients were excluded if they were diagnosed with rheumatoid arthritis or another inflammatory metabolic arthritis; had a history of hypersensitivity to HA; had a knee infection or localized skin disease at the site to be injected; had clinically apparent tense effusion of the target knee; had chronic pain anywhere requiring analgesic therapy that would confound the measurement of pain in the target knee; had received a corticosteroid injection within 24 weeks or oral corticosteroids within 12 weeks prior to screening; had an intra-articular HA within 36 weeks prior to screening or glucosamine–chondroitin within the past 3 months prior to baseline; had taken non-selective NSAIDs 48 h before baseline or selective NSAIDs up to 7 days before baseline; had a documented history of serious injuries to the target knee restricting ambulation; therapeutic arthroscopy and/or other surgical procedures within the 12 months prior to screening (diagnostic arthroscopy ≥ 60 days prior to screening was allowed); had a knee replacement; had a history of chondrocalcinosis, depression, or a sleep disorder; had a body mass index ≥ 39 kg/m^2^ or any other condition that could affect the outcome assessment.

This study was conducted in accordance with the ethical standards of committees on human experimentation (institutional and national) and the Declaration of Helsinki (1975, revised 2000). This study also adhered to Good Clinical Practice standards according to the International Council for Harmonisation and the International Organization for Standardization. The study protocol was approved by the relevant ethics committees at each participating institution. All patients provided written informed consent.

### 2.2. Study Design, Treatments, and Blinding

This was a prospective, active-controlled, randomized, parallel-group, double-blind, multicenter study conducted from January 2021 to December 2021 collaboratively at 14 study sites across the Czech Republic, Germany, and Poland. This trial is registered on ClinicalTrials.gov under the identifier NCT04732793.

After consent was obtained, study eligibility was confirmed and participants were randomized at each study site in a 1:1 ratio using an interactive web response system to Hyruan ONE (test group) or Durolane (comparator group) and stratified by side(s) of knee OA (unilateral or bilateral). Both participants and evaluators were blinded to which product was given. To maintain blinding, patients wore an eye-mask when they were injected. A separate unblinded investigator performed the injections, while a blinded investigator, called the “evaluator” (not present at any of the injections), performed all the assessments. All other study-site personnel were blinded.

The study consisted of six visits: visit 1, screening; visit 2 (baseline), administration of injection; visit 3, week 2; visit 4, week 8; visit 5, week 13; and visit 6 (end of study), week 26. Patients who withdrew from the study had an early termination visit. At screening, radiologic examination was assessed according to the Kellgren–Lawrence grade system [27], and patients were enrolled in the study if they were assessed as having Grade II–III OA in the 12 months prior to visit 1. The evaluator performed a physical assessment of the target knee by measuring the level of swelling and tenderness using a 4-point scale.

Each item of the WOMAC–Likert Index was assessed and the sub-scores on the scales of Pain, Stiffness, Physical Function, and the aggregate Total score were recorded [28,29]. Patient global assessment (PGA) was used to record the highest level of pain experienced, and investigator global assessment (IGA) was also utilized (both tests use a 100 mm visual analogue scale). A physical assessment of the target knee for swelling and tenderness on pressure was also performed at each visit. Vital signs (blood pressure, pulse rate, body temperature, and respiratory rate) were measured during the screening visit, prior to injection, and at each study visit. Laboratory tests, including C-reactive protein and erythrocyte sedimentation rate measurements, were performed at screening and at week 13, or at the early study termination visit.

Adverse events (AEs) or other diseases that occurred during the study could be treated using drugs that were not on the prohibited list. The only allowed rescue medication was acetaminophen at a maximum of 4 g within 24 h. Prohibited were: systemic steroids, chronic analgesics, and non-selective and selective NSAIDs that could affect study assessments; physical therapy (e.g., electrotherapy, cold therapy, laser, ultrasound, electrical stimulation); other alternative treatments for pain relief (e.g., acupuncture, cupping therapy, moxa treatment); anticoagulant agents (other than aspirin ≤ 325 mg/day for thromboprophylaxis or cardiovascular disease); and analgesics including acetaminophen for 48 h prior to study visits.

AEs were reported by investigators or spontaneously by patients, and were coded according to the *Medical Dictionary for Regulatory Activities* (MedDRA, version 24.1).

### 2.3. Outcome Measures

The primary endpoint was the change in the WOMAC–Likert Pain sub-scores from baseline to 13 weeks after HA injection.

The secondary endpoints were changes in the WOMAC–Likert Pain sub-score from baseline to week 26 post-injection; changes in the Total score and the Physical Function and Stiffness sub-scores from baseline to 13 and 26 weeks post-injection; changes in PGA and IGA scores from baseline to 13 and 26 weeks post-injection; the percentage of patients taking rescue medication at each visit post-injection; the level of swelling and tenderness of the target knee using a 4-point scale (0 = none, 1 = mild, 2 = moderate, 3 = severe); and the Outcome Measures in Rheumatology Clinical Trials–Osteoarthritis Research Society International (OMERACT-OARSI) responder rate [30] at 13 and 26 weeks post-injection. Criteria for an OMERACT-OARSI positive response were a WOMAC–Likert Pain or Physical Function sub-score improvement of ≥50% and ≥20 points over baseline or ≥20% and ≥10-point improvement over baseline in two of the following three measures: WOMAC–Likert Pain sub-score, WOMAC–Likert Physical Function sub-score, and PGA score.

The safety endpoints were the assessment of all AEs, vital signs, laboratory tests, and local reactions. AEs were classified as follows: (1) anticipated AE, (2) serious AE, (3) adverse device effect (ADE), (4) serious ADE (SADE), (5) anticipated SADE, (6) unanticipated SADE, or (7) device deficiency-related AE.

### 2.4. Statistical Methods

The non-inferiority margin was 1.6 with a standard deviation (SD) of 4. This was calculated based on 8% of 20, because 20 is the highest subscale score on the WOMAC–Likert Pain subscale, as determined from previous studies [31,32]. Therefore, 99 evaluable participants were needed in each group to have a statistical power of 80% with a one-sided type I error rate of 2.5%. With a 30% dropout rate assumed, a total of 284 participants (142 per group) were required.

The study had three analysis population sets: (1) safety set—all patients injected with either study device; (2) full analysis set (FAS)—all randomized patients injected with either study device and who had any follow-up data after injection at week 0 (visit 2) for the primary effectiveness assessment; and (3) per protocol set (PPS)—all patients included in the FAS who had no important protocol deviations, which were defined as protocol deviations that affected the effectiveness assessment, and completed the primary efficacy assessment at week 13 (visit 5). The PPS was used for the primary efficacy analysis.

To demonstrate non-inferiority of the study test product to an active comparator, the difference from baseline to 13 weeks post-injection in the WOMAC–Likert Pain sub-score was tested between groups in the PPS. An analysis of covariance (ANCOVA) model was constructed using main effects of treatment, baseline score, and side(s) of knee OA (unilateral, bilateral) as covariates. Adjusted means (least squares mean and standard error of the mean) by treatment and an estimate of the difference between adjusted means were calculated (test minus comparator). A 95% two-sided confidence interval (CI) based on the ANCOVA model was computed for the difference between the groups. If the upper limit of the CI was less than the non-inferiority margin of 1.6, then non-inferiority was demonstrated.

Between-group differences were analyzed for secondary efficacy endpoints as follows. For continuous variables, comparisons were made using the same ANCOVA model used to evaluate the primary efficacy outcome, and for categorical variables, comparisons were made using chi-squared tests (if at least 80% of the expected frequencies ≥ 5) or Fisher’s exact test (all other cases).

## 3. Results

### 3.1. Patients

**Figure 1:**
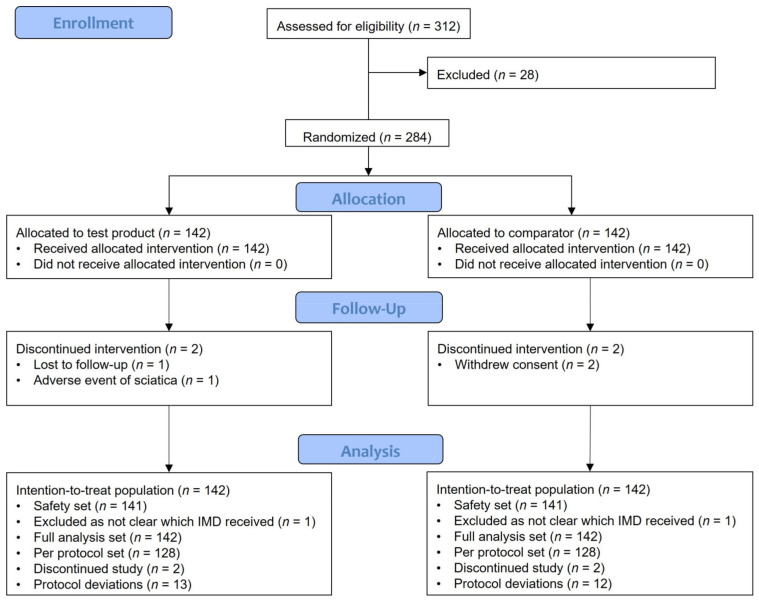
Flowchart illustrating the number of patients included in the study. IMD, investigational medical device.

The study flowchart is shown in Figure 1. Of the 312 patients screened, 284 were randomized 1:1 across each group. A total of 280/284 patients (98.6%) completed the study. Four patients (4/142 [2.8%]) discontinued the study: from the test group, one patient was lost to follow-up and another had an AE of sciatica that was unrelated to treatment; from the comparator group, two patients withdrew their consent.

The characteristics of the patients are shown in Table 1. Overall, the patient population was well balanced between the groups. All enrolled patients were White. The mean (SD) age was 62.6 (9.47) years, and the mean (SD) body mass index was 30.1 (4.17) kg/m^2^. The mean (SD) time since OA diagnosis for the total study population was 7.4 (6.53) years and was similar between the test and comparator groups (6.9 [6.07] years vs. 8.0 [6.94] years, respectively). A majority of patients had bilateral OA, and this was similar between groups (test group, 87/142 [61.3%]; comparator group, 88/142 [62.0%]).

A majority of patients (227/284 [79.9%]) had moderate OA as per radiographic evidence, with 118/142 (83.1%) in the test group and 109/142 (76.8%) in the comparator group. A total of 148/284 patients (52.1%) had Kellgren–Lawrence Grade II OA (74/142 [52.1%] in each group). Kellgren–Lawrence Grade III OA was present in 136/284 (47.9%) patients (68/142 [47.9%] in each group).

### 3.2. Outcomes

#### 3.2.1. Efficacy

**Figure 2:**
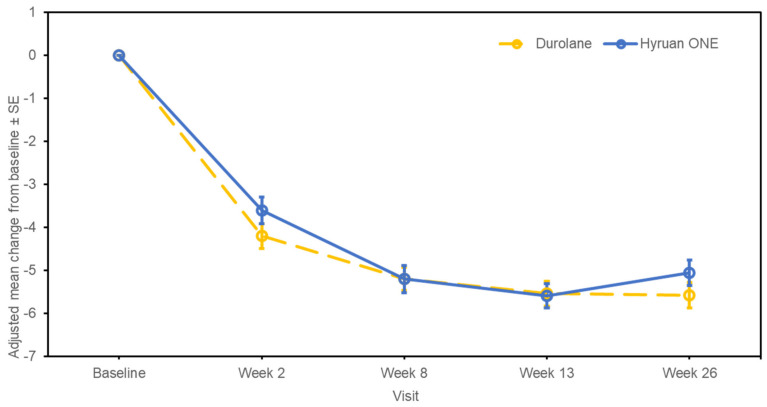
Mean changes from baseline to post-injection at all visits on the Western Ontario and McMaster Universities–Likert Pain sub-score (per-protocol set). SE, standard error.

The changes in WOMAC–Likert Pain sub-score from baseline to week 13 post-injection, which was the primary endpoint, are shown in Figure 2. The change in the WOMAC–Likert Pain sub-score at week 13 had an estimated adjusted mean difference between the test product and the comparator (standard error [SE]; 95% CI for difference) of −0.05 (0.398; −0.838, 0.729), with the upper limit of the CI less than the stated non-inferiority margin of 1.6, indicating non-inferiority of the test product. The sensitivity analysis performed on the FAS showed similar results, with an estimated adjusted mean difference between the two groups of −0.14 (0.390; −0.905, 0.629).

The outcomes of the sub-group analysis performed for the WOMAC–Likert Pain sub-score from baseline to 13 weeks post-injection showed similar results between both groups for the subgroups of side(s) of the knee OA (unilateral, bilateral), Kellgren–Lawrence scale grade (II, III), and country (Czech Republic, Germany, Poland) (Table S1).

**Figure 3:**
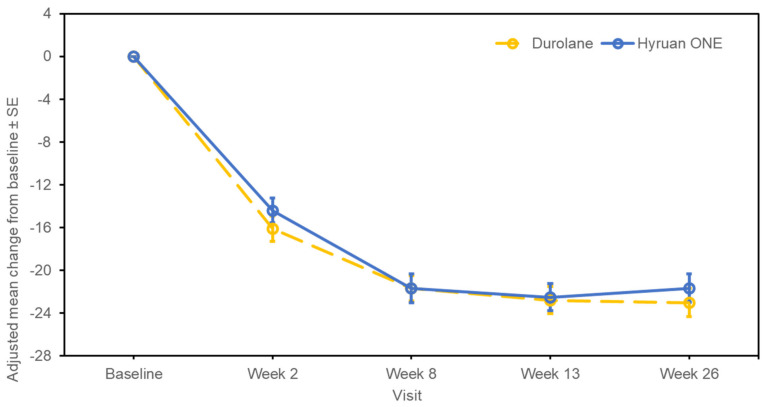
Western Ontario and McMaster Universities–Likert Total score changes from baseline to post-injection (per-protocol set). SE, standard error.

**Figure 4:**
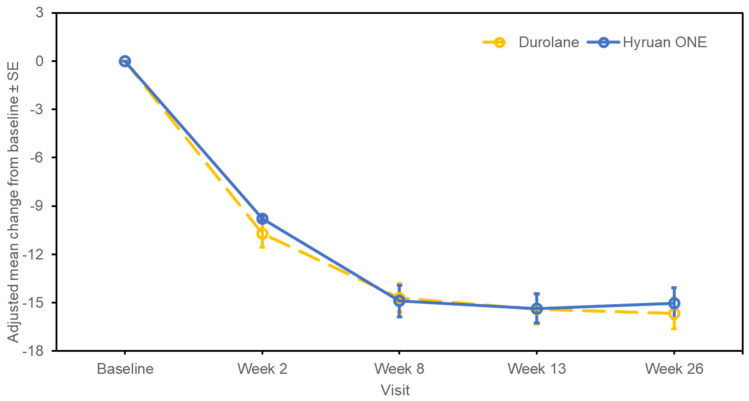
Western Ontario and McMaster Universities–Likert Physical Function sub-score changes from baseline to post-injection (per-protocol set). SE, standard error.

The changes in the WOMAC–Likert Pain sub-score from baseline to 26 weeks post-injection are shown in Figure 2. At week 26, the adjusted mean (SE; 95% CI) change from baseline was −5.06 (0.296; −5.645, −4.480) in the test group and −5.57 (0.295; −6.153, −4.992) in the comparator group. The estimated adjusted mean difference between the test product and the comparator (SE; 95% CI for difference) was 0.51 (0.413; −0.304, 1.324). For the changes in the WOMAC–Likert Total score, the adjusted mean (SE; 95% CI) change from baseline to week 26 was −21.67 (1.323; −24.276, −19.063) in the test group and −23.03 (1.319; −25.627, −20.431) in the comparator group (Figure 3). The estimated adjusted mean difference between the test product and the comparator (SE; 95% CI for difference) was 1.36 (1.848; −2.281, 5.000). The changes in the WOMAC–Likert Physical Function sub-score over the same time period are shown in Figure 4. At week 26, the adjusted mean (SE; 95% CI) change from baseline was −15.02 (0.951; −16.888, −13.143) in the test group and −15.66 (0.948; −17.524, −13.791) in the comparator group. The estimated adjusted mean difference between the test product and the comparator (SE; 95% CI for difference) was 0.64 (1.328; −1.974, 3.258). Furthermore, the changes in the WOMAC–Likert Stiffness sub-score, PGA score, and IGA score were also similar between the groups (Table S2). Physical assessments showed predominantly no or mild swelling or tenderness. Similar trends were observed between the groups (Table S3).

Less than 50% of all patients required rescue medication post-injection (test group, 61/128 [47.7%]; comparator group, 54/128 [42.2%]). The percentage of positive responders based on OMERACT-OARSI at week 13 was 64.8% (83/128) in the test group and 64.1% (82/128) in the comparator group, which decreased slightly at week 26 in both groups (57.8% [74/128] and 60.9% [78/128], respectively) (Table 2). The comparison between groups (95% CI for proportion difference, −0.152, 0.089) was not statistically significant (*p* = 0.61).

#### 3.2.2. Safety

A summary of the most common AEs in each group is shown in Table 3. In both groups, most events were mild to moderate, and severe AEs were rare. The number of patients experiencing treatment-emergent AEs (TEAEs) was similar between groups, with 43/141 (30.5%) patients experiencing 62 TEAEs in the test group and 47/141 (33.3%) patients experiencing 75 TEAEs in the comparator group. There were no fatal events or serious events related to the study device or injection procedure reported in either group. Most TEAEs were mild (28/141 patients [19.9%] with 35 events) in the test group, whereas in the comparator group, most TEAEs were either mild (27/141 patients [19.1%] with 37 events) or moderate (26/141 patients [18.4%] with 35 events). In total, 48 TEAEs recovered/resolved in 36/141 (25.5%) patients in the test group whereas 64 TEAEs recovered/resolved in 43/141 (30.5%) patients in the comparator group.

Arthralgia was the most common TEAE by preferred term in both groups (test group, 5/141 [3.5%] patients with six events; comparator group, 7/141 patients [5.0%] with seven events). The other most common TEAE by preferred term was headache (test group, 5/141 [3.5%]; comparator group, 4/141 [2.8%]). Arthralgia was also the most common treatment-emergent ADE (test group, 2/141 [1.4%]; comparator group, 3/141 [2.1%]) (Table 3). The other ADEs were injection site pain, joint swelling, injection site joint effusion, dermatitis contact, mobility decreased, and osteoarthritis.

One local reaction of injection site pain was reported as an AE in 1/141 (0.7%) patients in the test group. Five local reactions were reported as AEs in 4/141 (2.8%) patients in the comparator group (injection site pain, *n* = 2; joint effusion, *n* = 2; joint swelling, *n* = 1).

Three serious TEAEs were reported: ligament rupture (1/141 [0.7%] patients) in the test group, and angina unstable and vascular headache (2/141 [1.4%] patients) in the comparator group. None of the serious TEAEs were considered related to the study device or to the injection procedure, and all were resolved.

## 4. Discussion

The findings of this study indicate that the primary efficacy analysis was robust and treatment, with the cross-linked sodium hyaluronate test product non-inferior to an active comparator when assessed at 13 weeks. There were no obvious differences in safety between the treatment groups, as determined by the number and characteristics of reported AEs. The AEs were mostly mild or moderate and were not related to the treatment in most cases. Furthermore, most AEs were resolved and none was fatal. The incidence and types of AEs between the two treatments were also similar. The study showed that patients perceived a reduction in symptoms, as determined using the WOMAC–Likert scale, which evaluates pain, stiffness, and physical function. Likewise, OMERACT-OARSI responses were mostly positive. Swelling and tenderness of the target knee when under pressure showed a tendency to improve compared with baseline. Taken together, we can assume that intra-articular injection with cross-linked sodium hyaluronate improves pain in European patients with OA and with no significant AEs.

Overall, our results are similar to those reported by Zhang et al. [31], who compared the safety and efficacy of two HA formulations in Chinese patients with knee OA, one of which was the comparator in our study. In that study, the change in WOMAC–Likert Pain sub-score from baseline at week 18 was −5.97 and the incidence rate of TEAEs was 47.4% in the Durolane group. In our study, the change in WOMAC–Likert Pain sub-score from baseline at week 13 was −5.54 and the incidence rate of TEAEs was 33.3% in the comparator group. In both studies, arthralgia was the most common treatment-related AE, and is known to be one of the most frequently reported AEs among patients who receive HA injections [33]. Some differences between the studies include the higher OMERACT-OARSI response at week 26 in the Zhang et al. study [31] vs. ours (93.8% vs. 60.9%), as well as a lower percentage of patients that required rescue medication (<17% and 42.2%). According to Zhang et al., the reduced need for rescue medication was considered to be because of ethnic differences among the Chinese populations being studied. The use of rescue medication in our study was similar to the study conducted in Germany that used two forms of HMW hyaluronan (hyaluronan produced by biological fermentation, 49.3%; avian-derived hyaluronan that used cross-linking, 81.9%) in the treatment of knee OA [32]. Additionally, the OMERACT-OARSI response at week 26 in our study was similar to that reported in the multi-national study of Durolane (62.8%) [33].

Hyruan ONE has been approved in South Korea since 2013, following the completion of a double-blind, randomized, multi-center, non-inferiority study comparing Hyruan ONE with a 3-week course of HA injection that was conducted in South Korea [22]. In 2014, the product achieved the Conformité Européenne marking. The present study is the first to demonstrate the efficacy and safety of the test product in a European population. The study design (e.g., study endpoints, assessment time points, and prohibited concomitant medication) and target population in terms of key eligibility criteria of the present study were similar to those of the pivotal South Korean study [22]. That study reported that with test product treatment, there was a change in WOMAC–Likert Pain sub-score from baseline to week 12 post-injection of −3.96, an OMERACT-OARSI response rate of >50%, significant improvement in global assessment and swelling and tenderness on target knee, and a requirement for rescue medication in 63.9% of patients. These findings are comparable to those reported in the present study. Thus, the effectiveness and safety of the test product have been reconfirmed in a European population.

This study had a few limitations. First, we did not compare a single HA injection with multiple HA injections in the European population. However, this was investigated in the South Korean study and confirmed in an Asian population [22], which, as noted above, had comparable results with our study for a single HA injection. Thus, we expect similar efficacy and safety results with a single HA injection when compared with multiple HA injections in a European population. A further limitation was the absence of a placebo arm to demonstrate the superiority of HA injection to placebo, as it would be unethical to leave patients untreated, even in the clinical trial setting, given that there are treatments available. We were, however, able to compare the test product with another HMW cross-linked HA, Durolane, which has a moderate-strength recommendation for the treatment of OA of the knee in the AAOS guidelines [19]. Such a comparison can be used to establish the efficacy and safety of the test product, as Durolane has been injected over 2 million times worldwide [34] and is considered clinically safe, with a serious AE rate of 1.7% in clinical studies [31]. A third limitation was that our study followed patients for a maximum of 26 weeks (6 months). Data from recent meta-analyses suggest that the effects of intra-articular HA injections in relieving pain and improving joint function can endure for up to 6 months [10,18,35,36,37,38,39]. Cross-linked HAs are known to have increased residence time within the joint compared with non-chemically modified linear HAs [7], and therefore it would be interesting to investigate the longer-term effects of a single-dose HA in a follow-up study of its duration of effect. Despite these limitations, this is the first study of the test product outside of Asia, and importantly confirms its efficacy and safety in a European population. Additionally, the test product demonstrated similar efficacy and safety to a currently marketed cross-linked sodium hyaluronate product (Durolane). Thus, we consider our findings to be meaningful.

## 5. Conclusions

This post-market approval study met its primary endpoint and showed that treatment with cross-linked sodium hyaluronate with our test product was non-inferior to an active comparator in terms of reducing pain scores. Overall, our findings show that cross-linked sodium hyaluronate has favorable efficacy and safety in the treatment of European patients with mild-to-moderate OA of the knee.

## Figures and Tables

**Table 1:** Demographic characteristics (full analysis set).

Parameter	Test Group (*n* = 142)	Comparator Group (*n* = 142)	Overall (*n* = 284)
Sex, *n* (%)			
Male	53 (37.3)	52 (36.6)	105 (37)
Female	89 (62.7)	90 (63.4)	179 (63.0)
Age (years)			
Mean (SD)	62.7 (9.53)	62.5 (9.43)	62.6 (9.47)
Age-group (years), *n* (%)			
<65	76 (53.5)	78 (54.9)	154 (54.2)
≥65	66 (46.5)	64 (45.1)	130 (45.8)
Race, *n* (%)			
White	142 (100)	142 (100)	284 (100)
Country, *n* (%)			
Czech Republic	41 (28.9)	38 (26.8)	79 (27.8)
Germany	25 (17.6)	25 (17.6)	50 (17.6)
Poland	76 (53.5)	79 (55.6)	155 (54.6)
Body mass index (kg/m^2^)			
Mean (SD)	29.9 (4.28)	30.2 (4.06)	30.1 (4.17)
Kellgren–Lawrence grade of the target knee, *n* (%)			
Grade I	0	0	0
Grade II	74 (52.1)	74 (52.1)	148 (52.1)
Grade III	68 (47.9)	68 (47.9)	136 (47.9)
Grade IV	0	0	0
Time since OA diagnosis (years)			
Mean (SD)	6.9 (6.07)	8.0 (6.94)	7.4 (6.53)
Side of knee OA at screening, *n* (%)			
Unilateral	55 (38.7)	54 (38.0)	109 (38.4)
Bilateral	87 (61.3)	88 (62.0)	175 (61.6)

OA, osteoarthritis; SD, standard deviation.

**Table 2:** Responder rates by OMERACT-OARSI (per-protocol set).

Responder Rates by OMERACT-OARSI	Test Group (*n* = 128) *n* (%)	Comparator Group (*n* = 128) *n* (%)
Week 13	83 (64.8)	82 (64.1)
Comparison between groups		
95% CI for proportion difference	−0.109, 0.125	
χ^2^ *p*-value	0.8961	
Week 26	74 (57.8)	78 (60.9)
Comparison between groups		
95% CI for proportion difference	−0.152, 0.089	
χ^2^ *p*-value	0.6107	

CI, confidence interval; OMERACT-OARSI, Outcome Measures in Rheumatology Clinical Trials–Osteoarthritis Research Society International; χ^2^, chi-squared test. Missing data up to week 26 post-injection were addressed by considering patients with a missing OMERACT-OARSI response efficacy endpoint regardless of adherence to treatment or early discontinuation.

**Table 3:** Adverse events (safety set).

	Test Group (*n* = 141) *n* (%) Events	Comparator Group (*n* = 141) *n* (%) Events
TEAEs *	43 (30.5) 62	47 (33.3) 75
TEAE severity		
Mild	28 (19.9) 35	27 (19.1) 37
Moderate	18 (12.8) 25	26 (18.4) 35
Severe	2 (1.4) 2	3 (2.1) 3
Serious TEAEs	1 (0.7) 1	2 (1.4) 2
TEAEs with local reactions	1 (0.7) 1	4 (2.8) 5
TEAEs by preferred term ^†^		
Arthralgia	5 (3.5) 6	7 (5.0) 7
Headache	5 (3.5) 7	4 (2.8) 4
ADEs	5 (3.5) 6	8 (5.7) 12
ADEs by preferred term		
Arthralgia	2 (1.4) 2	3 (2.1) 3
Injection site pain	2 (1.4) 2	2 (1.4) 2
Joint swelling	1 (0.7) 1	3 (2.1) 3
Injection site joint effusion	0	1 (0.7) 2
Dermatitis contact	0	1 (0.7) 2
Mobility decreased	1 (0.7) 1	0
Osteoarthritis	0	1 (0.7) 1

ADE, adverse device effect (includes both device-related and injection procedure-related ADEs); TEAE, treatment-emergent adverse event. Any missing severity, relationship, or outcome was not imputed, but was classified as missing. Patients with more than one TEAE in a particular category (system organ class/preferred term) were counted only once in that category. * TEAEs were any events not present before the study device injection or any events already present that worsened in either intensity or frequency after the study device injection. ^†^ Occurring in ≥3% of patients.

## Data Availability

The data that support the findings of this study are available from the corresponding author upon reasonable request.

## Supplementary Materials

The following supporting information can be downloaded at https://www.mdpi.com/article/10.3390/jcm12082982/s1. Table S1: Change in WOMAC–Likert Pain sub-score from baseline to 13 weeks post-injection (per protocol set); Table S2: Changes in the Western Ontario and McMaster University (WOMAC)–Likert Stiffness sub-score, Patient Global Assessment score, and Investigator Global Assessment score from baseline to 26 weeks post-injection (per protocol set); Table S3: Test group and comparator group post-injection physical assessments of the target knee according to baseline values (per protocol set, *n* = 128).
