# Supplementary material for: Efficacy and Safety of Intra-Articular Cross-Linked Sodium Hyaluronate for the Treatment of Knee Osteoarthritis: A Prospective, Active-Controlled, Randomized, Parallel-Group, Double-Blind, Multicenter Study
Source: J Clin Med. 2023 Apr 19;12(8):2982. doi: 10.3390/jcm12082982 (PMC10145240; doi:10.3390/jcm12082982)
Supplement: Supplementary file 1 [file jcm-12-02982-s001.zip › jcm-2298750-supplementary.pdf]

Supplementary Materials

Efficacy and safety of intra-articular cross-linked sodium hyaluronate for the treatment of knee osteoarthritis: a prospective, active-controlled, randomized, parallel-group, double-blind, multicenter study

Tomasz Blicharski, et al.

Table S1. Change in WOMAC-Likert Pain sub-score from baseline to 13 weeks post-injection (per protocol set)

	Kellgren–Lawrence scale		Side of knee OA		Country		
	Grade II	Grade III	Unilateral	Bilateral	Czech Republic	Germany	Poland
**Test Group**							
**Baseline**							
n	67	61	49	79	38	20	70
**Mean (SD)**	10.6 (1.87)	10.9 (1.73)	10.4 (1.74)	10.9 (1.82)	10.5 (1.67)	10.9 (2.08)	10.8 (1.80)
**Median (Min, Max)**	10.0 (7, 15)	11.0 (8, 16)	10.0 (7, 15)	11.0 (7, 16)	10.0 (7, 14)	10.0 (8, 16)	11.0 (7, 15)
**Week 13**							
n	67	61	49	79	38	20	70
**Mean (SD)**	5.2 (2.83)	5.1 (3.01)	5.2 (2.83)	5.2 (2.97)	4.8 (2.72)	6.4 (3.91)	5.0 (2.62)
**Median (Min, Max)**	5.0 (0,13)	5.0 (0, 15)	5.0 (0, 15)	5.0 (0, 14)	5.0 (0, 13)	5.5 (0, 15)	5.0 (0, 10)
**Change from Baseline**							
n	67	61	49	79	38	20	70
**Mean (SD)**	−5.4 (3.14)	−5.8 (2.72)	−5.2 (2.95)	−5.7 (2.94)	−5.7 (2.79)	−4.5 (2.96)	−5.8 (2.98)
**Median (Min, Max)**	−5.0 (−11, 1)	−6.0 (−12, 0)	−5.0 (−11, 1)	−6.0 (−12, 1)	−5.0 (−11, 1)	−4.0 (−12, 0)	−6.0 (−11, 1)
**Comparator Group**							
**Baseline**							
n	68	60	50	78	33	22	73

**Mean (SD)**	10.8 (1.89)	11.1 (1.87)	10.8 (1.97)	11.0 (1.83)	10.2 (1.62)	9.6 (1.68)	11.6 (1.73)
**Median (Min, Max)**	11.0 (7, 16)	11.0 (7, 15)	10.0 (7, 15)	11.0 (7, 16)	10.0 (7, 14)	9.0 (7, 13)	12.0 (7, 16)
**Week 13**							
n	68	60	50	78	33	22	73
**Mean (SD)**	5.2 (3.29)	5.5 (3.85)	5.4 (3.70)	5.3 (3.48)	5.0 (3.16)	5.4 (3.39)	5.5 (3.79)
**Median (Min, Max)**	5.0 (0, 13)	5.0 (0, 17)	5.0 (0, 17)	5.0 (0, 13)	4.0 (0, 13)	5.0 (0, 13)	5.0 (0, 17)
**Change from Baseline**							
n	68	60	50	78	33	22	73
**Mean (SD)**	−5.6 (3.61)	−5.6 (3.91)	−5.4 (4.10)	−5.7 (3.50)	−5.3 (3.38)	−4.2 (3.07)	−6.2 (3.98)
**Median (Min, Max)**	−5.0 (−15, 3)	−6.5 (−13, 8)	−6.0 (−15, 8)	−6.0 (−12, 3)	−6.0 (−12, 3)	−3.5 (−10, 2)	−7.0 (−15, 8)

OA, osteoarthritis; SD, standard deviation

Table S2. Changes in the Western Ontario and McMaster University (WOMAC)-Likert Stiffness sub-score, Patient Global Assessment score, and Investigator Global Assessment score from baseline to 26 weeks post-injection (per protocol set)

	Scores and changes from baseline to Week 26	
	**Test group**	**Comparator group**
**WOMAC-Likert Stiffness sub-score**		
**Baseline**		
n	128	128
**Mean (SD)**	4.1 (1.25)	4.0 (1.50)
**Median (Min, Max)**	4.0 (0, 7)	4.0 (0, 8)
**Week 26**		
n	123	123
**Mean (SD)**	2.5 (1.40)	2.2 (1.66)
**Median (Min, Max)**	2.0 (0, 6)	2.0 (0, 8)
**Change from baseline**		
n	123	123
**Mean (SD)**	−1.6 (1.48)	−1.8 (1.86)
**Median (Min, Max)**	−2.0 (−6, 2)	−2.0 (−7, 3)
**Estimated adjusted mean difference (SE; 95% CI for difference)**	0.21 (0.185; –0.159, 0.571)	
**Patient Global Assessment score**		
**Baseline**		
n	128	128
**Mean (SD)**	60.9 (15.30)	61.1 (14.23)
**Median (Min, Max)**	62.0 (15, 88)	62.0 (22, 92)
**Week 26**		
n	123	123
**Mean (SD)**	30.8 (21.96)	29.3 (22.31)
**Median (Min, Max)**	26.0 (0, 91)	26.0 (0, 90)
**Change from baseline**		
n	123	123
**Mean (SD)**	−30.0 (22.61)	−31.3 (23.51)
**Median (Min, Max)**	−28.0 (−81, 40)	−32.0 (−92, 36)
**Estimated adjusted mean difference (SE; 95% CI for difference)**	1.40 (2.733; –3.984, 6.784)	
**Investigator Global Assessment**		
**Baseline**		
n	128	128
**Mean (SD)**	54.7 (15.34)	55.0 (14.0)

**Median (Min, Max)**	54.50 (14, 89)	56.0 (14, 87)
**Week 26**		
n	123	123
**Mean (SD)**	23.7 (16.07)	24.0 (17.01)
**Median (Min, Max)**	22.0 (0, 74)	20.0 (0, 80)
**Change from baseline**		
n	123	123
**Mean (SD)**	−30.6 (20.57)	−30.4 (20.26)
**Median (Min, Max)**	−29.0 (−79, 26)	−32.0 (−79, 21)
**Estimated adjusted mean difference (SE; 95% CI for difference)**	–0.28 (2.090; –4.395, 3.840)	

CI, confidence interval; SD, standard deviation; SE, standard error

Table S3. Shift table of test group and comparator group post-injection physical assessments of the target knee according to baseline values (per protocol set, *n* = 128)

	Degree of symptoms at Week 26, *n* (%)									
	Test group					Comparator group				
**Degree of symptoms at baseline**	**None**	**Mild**	**Moderate**	**Severe**	**Total**	**None**	**Mild**	**Moderate**	**Severe**	**Total**
**Swelling**										
**None**	72 (56.3)	3 (2.3)	1 (0.8)	0	76 (59.4)	65 (50.8)	4 (3.1)	0	0	69 (53.9)
**Mild**	22 (17.2)	13 (10.2)	2 (1.6)	0	37 (28.9)	26 (20.3)	17 (13.3)	3 (2.3)	1 (0.8)	47 (36.7)
**Moderate**	1 (0.8)	9 (7.0)	4 (3.1)	0	14 (10.9)	4 (3.1)	6 (4.7)	1 (0.8)	1 (0.8)	12 (9.4)
**Severe**	0	1 (0.8)	0	0	1 (0.8)	0	0	0	0	0
**Total**	95 (74.2)	26 (20.3)	7 (5.5)	0	128 (100)	95 (74.2)	27 (21.1)	4 (3.1)	2 (1.6)	128 (100)
**Tenderness on pressure**										
**None**	21 (16.4)	0	0	0	21 (16.4)	25 (19.5)	0	1 (0.8)	0	26 (20.3)
**Mild**	17 (13.3)	8 (6.3)	3 (2.3)	0	28 (21.9)	10 (7.8)	11 (8.6)	2 (1.6)	0	23 (18.0)
**Moderate**	27 (21.1)	31 (24.2)	12 (9.4)	1 (0.8)	71 (55.5)	28 (21.9)	34 (26.6)	10 (7.8)	0	72 (56.3)
**Severe**	3 (2.3)	5 (3.9)	0	0	8 (6.3)	3 (2.3)	3 (2.3)	1 (0.8)	0	7 (5.5)
**Total**	68 (53.1)	44 (34.4)	15 (11.7)	1 (0.8)	128 (100)	66 (51.6)	48 (37.5)	14 (10.9)	0	128 (100)
